# Supplementary material for: Antibiotic resistance potential of the healthy preterm infant gut microbiome
Source: PeerJ. 2017 Jan 25;5:e2928. doi: 10.7717/peerj.2928 (PMC5270596; doi:10.7717/peerj.2928)
Supplement: Figure S1 — Pearson correlations of species relative abundance for the four replicates, including within run and across machine types. [file peerj-05-2928-s001.pdf]

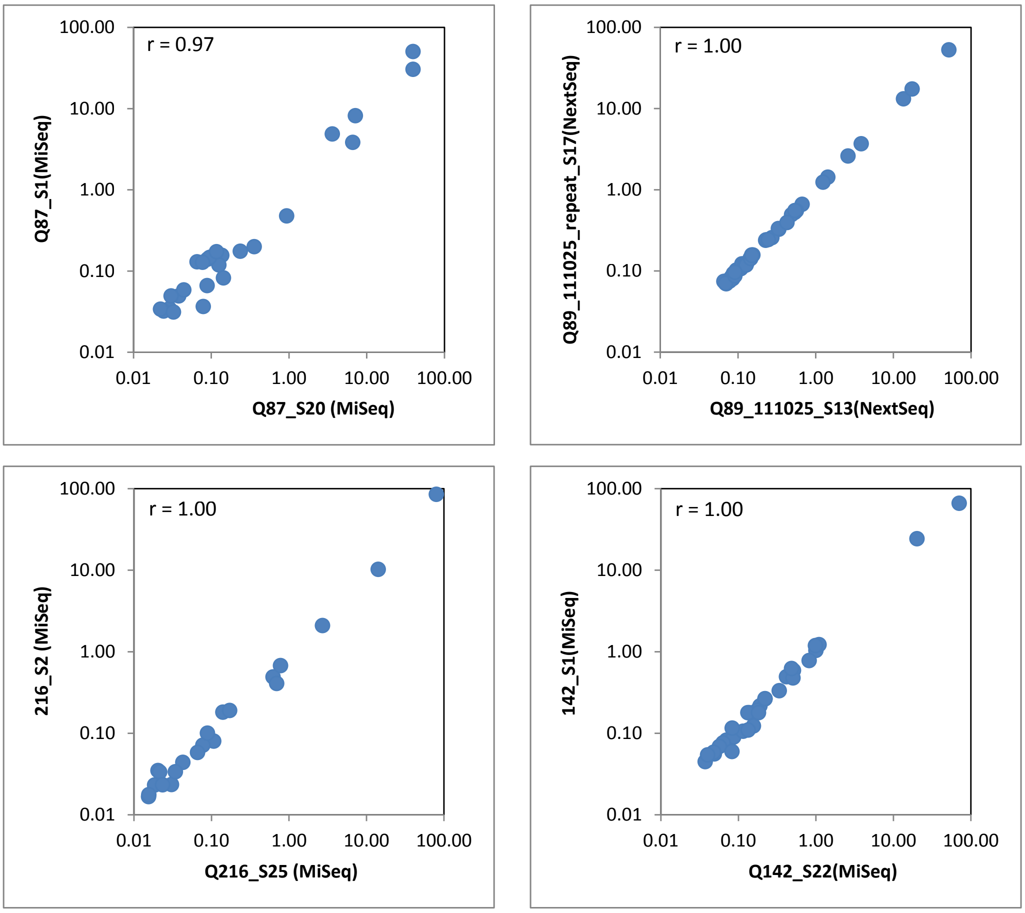

Figure S1. Correlation of replicates by species abundance. Pearson correlations of species relative abundance for the four replicates, including within run and across machine types.
