# Supplementary material for: Antibiotic resistance potential of the healthy preterm infant gut microbiome
Source: PeerJ. 2017 Jan 25;5:e2928. doi: 10.7717/peerj.2928 (PMC5270596; doi:10.7717/peerj.2928)
Supplement: Figure S2 — Diversity measured using the Shannon-Weaver index on left axis, and total antibiotic exposure in days on right axis. [file peerj-05-2928-s002.pdf]

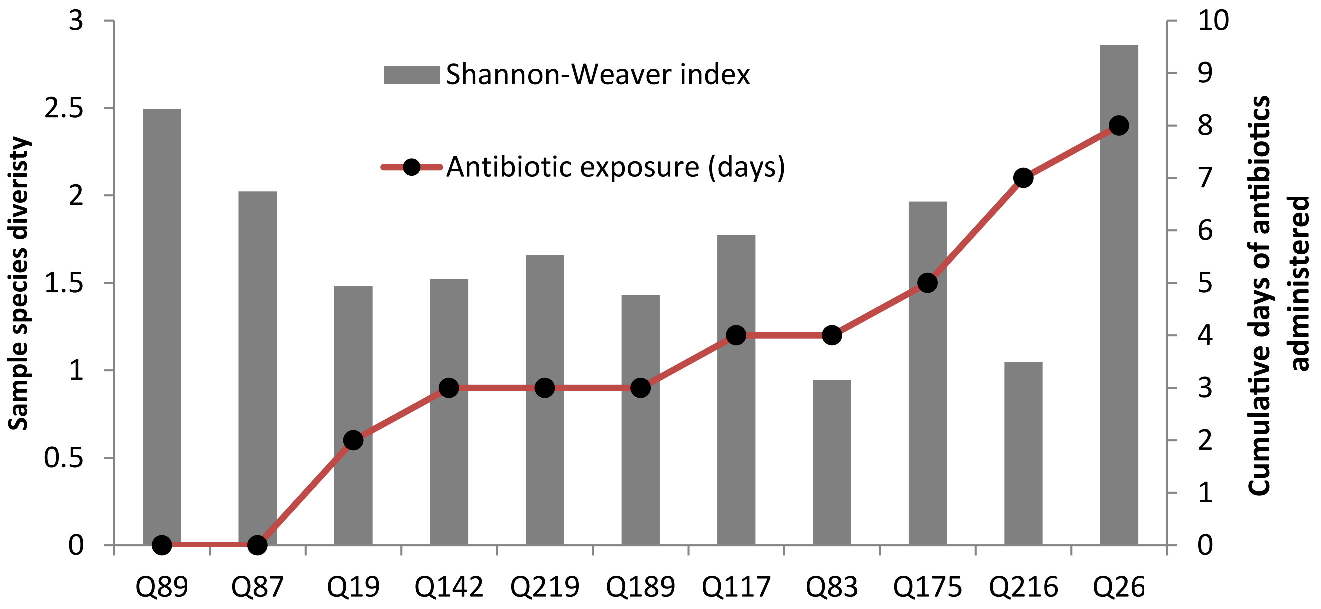

Figure S2. Metagenome diversity. Diversity measured using the Shannon-Weaver index on left axis, and total antibiotic exposure in days on right axis.
