# Supplementary material for: Antibiotic resistance potential of the healthy preterm infant gut microbiome
Source: PeerJ. 2017 Jan 25;5:e2928. doi: 10.7717/peerj.2928 (PMC5270596; doi:10.7717/peerj.2928)
Supplement: Figure S3 — Neighbor-joining tree built using estimated distances between a collection of 118 complete S. aureus genomes, and assembled S. aureus genomes from 4 preterm infant metagenomes. Genomes derived from the premature infant samples are highlighted in red. S. epidermidis used as the outlier, and this branch has been truncated for presentation purposes. Scale bar shows number of nucleotide substitutions per site. [file peerj-05-2928-s003.pdf]

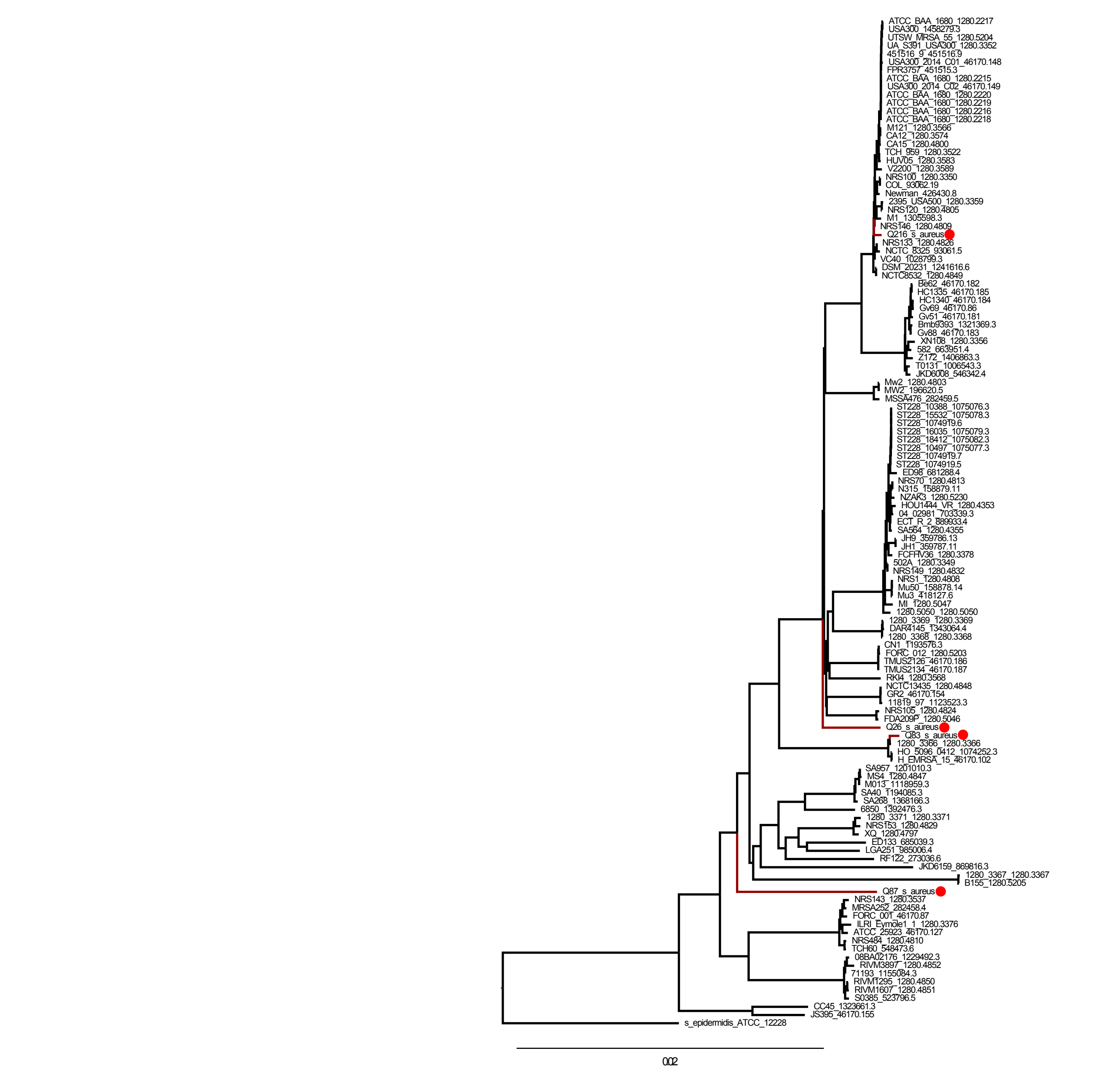

Figure S3. *S. aureus* phylogeny. Neighbor-joining tree built using estimated distances between a collection of 118 complete *S. aureus* genomes, and assembled *S. aureus* genomes from 4 preterm infant metagenomes. Genomes derived from the premature infant samples are highlighted in red. *S. epidermidis* used as the outlier, and this branch has been truncated for presentation purposes. Scale bar shows number of nucleotide substitutions per site.
